# Computing the adaptive cycle

**DOI:** 10.1038/s41598-020-74888-y

**Published:** 2020-10-23

**Authors:** Wolfgang zu Castell, Hannah Schrenk

**Affiliations:** 1 Helmholtz Zentrum München, Ingolstädter Landstraße 1, 85764 Neuherberg, Germany; 2 TUM München, Boltzmannstraße 3, 85748 Garching bei Munich, Germany

**Keywords:** Ecological networks, Computational science, Scientific data, Sustainability, Theoretical ecology

## Abstract

Gunderson’s and Holling’s adaptive cycle metaphor provides a qualitative description of the development of a dynamically evolving complex system. According to the metaphor, a complex system alternately passes through phases of stability and predictability and phases of reorganization and stochasticity. So far, there have been no attempts to quantify the underlying notions in a way which is independent of the concrete realization of the system. We propose a method which can be applied in a generic way to estimate a system’s position within the adaptive cycle as well as to identify drivers of change. We demonstrate applicability and flexibility of our method by three different case studies: Analyzing data obtained from a simulation of a model of interaction of abstract genotypes, we show that our approach is able to capture the nature of these interactions. We then study European economies as systems of economic state variables to illustrate the ability of system comparison. Finally, we identify drivers of change in a plant ecosystem in the prairie-forest. We hereby confirm the conceptual dynamics of the adaptive cycle and thus underline its usability in understanding system dynamics.

## Introduction

Understanding the dynamics of self-organizing systems is steadily gaining attention with the challenge to deal with increasing complexity and to manage systems on a sustainable basis . Examples range through many areas of high societal relevance, such as coping with climate change, managing economical crises, or adapting to the digital transformation. One of the major challenges in managing complex systems of interacting agents is to close the gap between qualitative theory describing system evolution and quantitative methods accessing the actual state and development of a given system.

**Figure 1:**
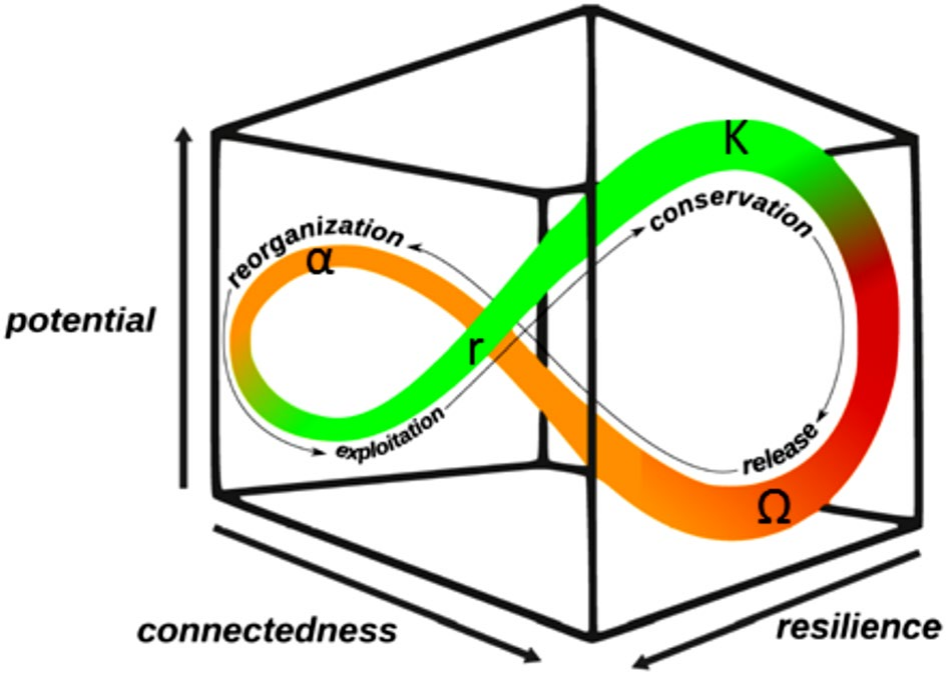
Visualization of Gunderson’s and Holling’s adaptive cycle.

Gunderson and Holling^1^ have provided a conceptual metaphor characterizing the evolution of evolving systems in terms of a cycle of four phases, each of which focusing on particular characteristic drivers of change. Hereby, the *adaptive cycle* is deduced from the interplay of three essential variables: *potential*, *connectedness* and *resilience*. Together, these span a space within which system development is depicted by the characteristic ’lying-eight’ figure (see Fig. 1). Using these coordinate variables, four phases of system development can be identified. The consecutive phases of *exploitation* and *conservation* are characterized by rather stabilizing conditions, with predictability guiding system behaviour. In contrast, the following *release* and *reorganization* phases lead to (partial) system breakdown and reorientation. Stochasticity of information sensed from the environment dominates, forcing the system to make use of its adaptive potential.

Given that the position of a system within the adaptive cycle can be estimated from data, the concept provides a powerful tool to better understand system behaviour, opening-up opportunities for sustainable system management and control within a dynamic environment.

In spite of its immediate comprehensibility, the adaptive cycle so far has to a large extent resisted any attempt to be captured in terms of quantitative measurements. Advances have been presented studying urban systems^2^, phytoplankton communities^3^, public governance of land use^4^, or coastal-marine socio-ecological systems^5^. Besides, systematic assessments of the concept have been pursued^6,7^.

The major difficulty in quantifying the adaptive cycle lies in the abstract character of its defining variables, which need to be derived from quantifiable variables based on longitudinal measurements. Every one of the three characteristic variables represents a complex notion on its own which needs interpretation within the context of a concrete system. For example, resilience comprises differing aspects^8^ such as resistance or precariousness which might need to be estimated independently of each other.

It is our aim to operationalize the adaptive cycle independently of the underlying system and on the basis of commonly available data. Towards this end, we utilize concepts of information theory providing a generic way to estimate potential, connectedness and resilience from time series data. Our approach can therefore also be used to compare various systems regardless of their specific instantiation. Making the adaptive cycle quantitatively accessible is crucial for driving a deeper scientific assessment of this metaphorical concept and shedding light on its underlying driving forces. Herein lies the central motivation for the present work.

Starting from the central assumption that any effective interaction among two agents of a system must lead to a transfer of information among them, we propose to utilize *transfer entropy*^9^ to quantify interaction. Doing so, we can derive a network of transfers of entropy, providing a quantitative model of the system which

(i) is based solely on the concept of interaction,
(ii) can be estimated from any kind of time series data, reflecting the outcome of interaction among agents, and
(iii) provides a basis for quantifying potential, connectedness and resilience, independently of the concrete instantiation of the system.

Our approach thus can be used in a generic way to assess system development over time, identify driving forces, or simply compare systems.

After presenting the approach and its implementation, we provide an example based on a simulation of the Tangled Nature Model (TNM)^10^. The TNM models a system of interacting ‘genotypes’ and has been shown to generate emergent patterns of stability followed by periods of change purely based on interaction. As a second example, we apply our approach to economic data for three European countries, demonstrating the power of the approach in explicitly comparing different national economies. In order to demonstrate the ability of the proposed method in analysing drivers of system development, we provide a third example of a plant ecosystem being managed under a defined intervention scheme.

## Methods

Our method is based on the assumption that the information structure of a system captures every effective interaction among its agents and thereby reflects the condition of the system. The abstract nature of information theory allows us to analyse systems independently of their specific instantiation. We only rely on the availability of longitudinal data reflecting the strength of the system’s individual components in a very broad sense. Hence, in general, our method can be applied to any complex system. The only condition is that for a given period of time and for every component of the system, a time series of quantitative data reflecting the outcome of interactions exists. Such time series could exemplarily be biomass of a plant species, number of individuals of an animal species, or sales of a company. The data type can differ among the components of the system, i.e. be heterogeneous.

In a first step, networks of information transfer are inferred via pairwise estimation of *transfer entropy*^9^ among all agents. Considering these networks and, in particular, their development over time, offers insights into functional interactions.

In the second step, potential, connectedness and resilience are computed solely using the networks of information transfer (see Supplementary A for a review of the adaptive cycle and its defining variables). Here, we utilize *capacity* and *ascendency* as being defined by Ulanowicz in the context of ascendency theory^11^. Note that Ulanowicz also used information theory to define capacity as an entropy of flows and ascendency as mutual information between inflow and outflow. While the first one is a measure of the average indeterminacy in the fluxes of the network, the latter quantifies the efficiency the system has in making use of its capacity^12^. However, being rooted in systems ecology, Ulanowicz always considered flows of physical quantities, such as energy or resource fluxes. In contrast, we will derive the quantities from networks of information transfer, abstracting from the physical representation of the interaction. Thus, potential is the capacity of the network of information transfer, and connectedness the corresponding ascendency.

The challenging part of our approach is to find an appropriate measure of resilience. There exist various conceptions and following definitions of resilience^13^. For our purposes, Holling’s definition of resilience fits best, namely to define resilience as ”*the magnitude of disturbance that can be absorbed before the system changes the variables and processes that control behavior*” (Ref.^1^, p. 28). There have been various approaches to make this notion measurable, however, all of them either depending on the specific system under observation^14–17^ or requiring deep knowledge of the system dynamics^18^. Resilience has also been studied from a network perspective (see e.g.^19,20^). Since we are modeling complex systems as networks of information transfer, our definition is inspired by a common concept in spectral graph theory. We use the so-called graph Laplace operator, which captures vulnerability of a network with respect to perturbations of its topological structure.

Taken together, the development of these three variables displays the system’s course through the adaptive cycle, helping to better understand system maturation and to evaluate its current condition. We will now provide a detailed description of our method, its implementation, and its application in the three case studies presented in this paper.

### Step 1: estimation of networks of information transfer

Let $\mathscr {V}$ be a collection of variables, quantifying the state of agents defining a system. Let $I = (i_1, \dots , i_N)$ and $J = (j_1, \dots , j_N)$ be two sets of samples of states for the components *I* and *J*, say. For example, *I* and *J* can be identified with abundances of two interacting species at time points $1, \dots , N$. We consider the time series *I* and *J* as realisations of two approximately stationary discrete Markov processes. This allows us to compute Schreiber’s transfer entropy^9^, serving as a measure of their effective interaction. Transfer entropy from *J* to *I* is defined as

$$\begin{aligned} T_{J \rightarrow I} = \sum _{n = 1}^{N-1} p \left( i_{n+1},i_{n}, j_{n} \right) \cdot \log \left( \frac{p \left( i_{n+1}|i_{n}, j_{n} \right) }{p \left( i_{n+1} | i_{n} \right) } \right) . \end{aligned}$$

$T_{J \rightarrow I}$ quantifies the average reduction in uncertainty about the future of *I* given the past of *J*. In other words, how much additional information do we gain about the next state of *I*, knowing not only the past of *I* itself, but the past of *J* as well. In the literature, a multitude of studies on the interpretation of transfer entropy in general and in specific contexts can be found^21–23^.

As the probabilities occurring in the definition of transfer entropy are in general not known, we have to estimate transfer entropy on the basis of given realizations of the random variables, i.e. the data given as samples of the time series. Typically, we do not use all available samples to estimate transfer entropy at time *t* but samples falling within a certain window of time preceding time *t*. The size $w_t$ of this windows can either be fixed, or depend on the time *t*, e.g. $w_t = t$. In the first case, the window is “shifted” going along with *t* to guarantee transfer entropy always being estimated on the same number of samples. In the second case, the window starts at the beginning of the time series and is extended with increasing *t*. In this case, the full history of the time series is considered for estimating transfer entropy. The choice of the window size depends on the system under consideration. In any case, it should be at least as large as the assumed order of the underlying Markov process. We then compute the information transfer from *J* to *I* at time *t* estimating transfer entropy over the period $t-w_t+1,\dots ,t$. To be precise,

$$\begin{aligned} T_{J \rightarrow I}^t = \sum _{n = t-w_t+1}^{t} p \left( i_{n+1},i_{n}, j_{n} \right) \cdot \log \left( \frac{p \left( i_{n+1}|i_{n}, j_{n} \right) }{p \left( i_{n+1} | i_{n} \right) } \right) . \end{aligned}$$

Depending on the size of $w_t$ and the data being available, it can be useful to increase the number of data points falling within every window by interpolation. For our calculations, we used the Matlab function pchip. Interpolation stabilizes the estimation in case of small window sizes. At the same time, interpolating too many points reduces stochasticity in the time series due to the deterministic component being introduced by the interpolation model. Thus, there is a trade-off between stochasticity and stability which has to be taken into account.

We estimated $T_{J \rightarrow I}^t$ using the Kraskov-Stögbauer-Grassberger (KSG) estimator TransferEntropyCalculatorKraskov as being provided with the JIDT toolkit^24^. For all our calculations, the function has been called using the data $(j_{t-w_t+1},\dots ,j_t)$ and $(i_{t-w_t+1},\dots ,i_t)$ in the mode computeAverageLocalOfObservations. For all other parameters of the estimation procedure, we used the default values $k=k_{tau}=l=l_{tau}=delay=1$. Other choices of these parameters can be reasonable depending on the specific system to be analysed. To distinguish actual interactions from random noise, we tested all estimates via hundred-fold bootstrapping using the function computeSignificance(100) incorporated in the toolkit. Tests passing below a given significance level have been accepted and thus lead to an edge between the corresponding components with the estimated transfer entropy defining the corresponding weight. Estimating and testing for all pairs of components at fixed time *t*, we finally obtained a weighted, directed graph

$$\begin{aligned} G^t = \left( \mathscr {V},\{T_{J \rightarrow I}^t|(J,I) \in \mathscr {V} \times \mathscr {V} \} \right) \end{aligned}$$

as being our inferred model of interaction at time *t*. Given time series of abundances of length *N* for each component, this results in a sequence of interaction networks for time points $w_1,\dots ,N$.

Summarizing, the first step infers models of interaction among the given variables in form of a series of networks capturing the interaction patterns and their strengths. These network models can then be used in the second step to actually determine the position of the system within the adaptive cycle.

### Step 2: determining potential, connectedness, and resilience

As mentioned before, our definitions of potential and connectedness are based on Ulanowicz’s notions of capacity and ascendency^11^. Ulanowicz provides further information on the theoretical background of these measures. Let

$$\begin{aligned} T^t = \sum _{(J,I) \in \mathscr {V} \times \mathscr {V}} T_{J \rightarrow I}^t \end{aligned}$$

be the total transfer of the system at time *t*. We further introduce the following shorthand notation

$$\begin{aligned} T_{J}^{\text {out},t} = \sum _{I \in \mathscr {V}} T_{J \rightarrow I}^t \qquad \text{ and } \qquad T_{I}^{\text {in},t} = \sum _{J \in \mathscr {V}} T_{J \rightarrow I}^t. \end{aligned}$$

Define

$$\begin{aligned} P^t = - \sum _{(J,I) \in \mathscr {V} \times \mathscr {V}} T_{J \rightarrow I}^t \cdot \log \left( \frac{T_{J \rightarrow I}^t}{T^t} \right) \qquad \hbox {as the system's} \,potential \,\hbox {at time} \,t \end{aligned}$$

and

$$\begin{aligned} C^t = \sum _{(J,I) \in \mathscr {V} \times \mathscr {V}} T_{J \rightarrow I}^t \cdot \log \left( \frac{T_{J \rightarrow I}^tT^t}{T_{J}^{\text {out,t}}T_{I}^{\text {in,t}}} \right) \qquad \,\hbox {as its} \,connectedness \,\hbox {at time}\, t. \end{aligned}$$

Being essentially a sum over the indeterminacy in each transfer within the system, potential can be interpreted as a measure of the system’s power for evolution and its ability to develop. Recall that development of the system as a whole necessarily relies on communication, i.e., transfer of information among its components. In contrast, connectedness measures the degree of internal coherence of the system by contrasting information leaving one component with information arriving at another component.

In order to define resilience, we need to capture vulnerability of the system with respect to unforeseen perturbation. In terms of graph theory, this can be achieved by studying the eigenvalues of a certain matrix, being associated with the graph. Indeed, the smallest non-trivial eigenvalue of the so-called *graph Laplacian* of an undirected graph quantifies the vulnerability of the graph with respect to disturbance of the topology of the graph^25,26^. In our case, we need to transfer this idea to the case of the directed graphs $G^t$.

Thus, given $G^t=\left( \mathscr {V},T^t_{J \rightarrow I}|(J,I) \in \mathscr {V} \times \mathscr {V}\right)$ be a non-empty, weighted, directed graph with vertex set $\mathscr {V}$. Let further $c > 0$ be a constant. Let $D_{out}$ and $D_{in}$ be the diagonal matrix of out-degrees and in-degrees, respectively, and *A* the weighted adjacency matrix. We then define the following Laplace type operators of $G^t$:

$$\begin{aligned} L_{out} = c \cdot D^{-\frac{1}{2}}_{out} \left( D_{out}- A \right) D^{-\frac{1}{2}}_{out}, \quad \hbox { and }\quad L_{in} = c \cdot D^{-\frac{1}{2}}_{in} \left( D_{in} - A \right) D^{-\frac{1}{2}}_{in}, \end{aligned}$$

following the convention that $D^{-\frac{1}{2}}_*(u,u) = 0$ for $D_*(u,u) = 0$. Note that, for the sake of readability, we omitted the superscript *t* in these definitions. For all case studies presented in this paper, we used

$$\begin{aligned} c = \frac{1}{\max \{ T^t_{J \rightarrow I}|(J,I) \in \mathscr {V} \times \mathscr {V} \}} \end{aligned}$$

as standardization constant.

Since *A* is no longer symmetric, the spectrum of $L_{out}$ and $L_{in}$ is complex in general. Nevertheless, the distance of the spectrum to the imaginary axis in the complex plane still determines the stability of the graph. Therefore, we define resilience of the graph *G* as the smallest, non-trivial absolute value of the real parts of all eigenvalues of its two Laplacian matrices, i.e.

$$\begin{aligned} R^t = \min \left\{ |\mathfrak {R}\sigma | :\sigma \in {{\,\mathrm{Spec}\,}}(L_{out}) \cup {{\,\mathrm{Spec}\,}}(L_{in}), \sigma \ne 0\right\} . \end{aligned}$$

See Supplementary E for a more detailed explanation motivating this definition as well as for an alternative definition of the involved Laplacian matrices.

Our definitions of the three systemic variables are summarized in Table 1. In addition, Table 2 displays basic information concerning the data sets and parameters of our case studies. Note that, for visualization purposes, Figs. 3, 4, and 5 show a smoothed version of the estimated variables as being obtained by applying the R functions smooth.spline and splinefun.

**Table 1:** Summary of the definitions of the three systemic variables.

Systemic variable	Definition
Potential	$P = - \sum _{(J,I) \in \mathscr {V} \times \mathscr {V}} T_{J \rightarrow I} \cdot \log \left( \frac{T_{J \rightarrow I}}{T} \right)$
Connectedness	$C = \sum _{(J,I) \in \mathscr {V} \times \mathscr {V}} T_{J \rightarrow I} \cdot \log \left( \frac{T_{J \rightarrow I}T}{T_{J}^{\text {out}}T_{I}^{\text {in}}} \right)$
Resilience	$R = \min \left\{ |\mathfrak {R}\sigma | :\sigma \in {{\,\mathrm{Spec}\,}}(L_{out}) \cup {{\,\mathrm{Spec}\,}}(L_{in}), \sigma \ne 0\right\}$

**Table 2:** Data and parameters of the presented case studies.

	Tangled nature model	Euro crisis	Kansas
System	Community of genotypes	European country	Plants community
Components	Genotypes	Economic variables	Plant species
Number of components	24	19	39
Type of abundance data	Number of individuals	Heterogeneous	Percentage of plot covered
Length of time series	5000	12	14
Window size $w_t$	100	*t* (starting at 7)	6
Interpolated window size	100	18	18
Significance level	0.01	0.01	0.05

**Figure 2:**
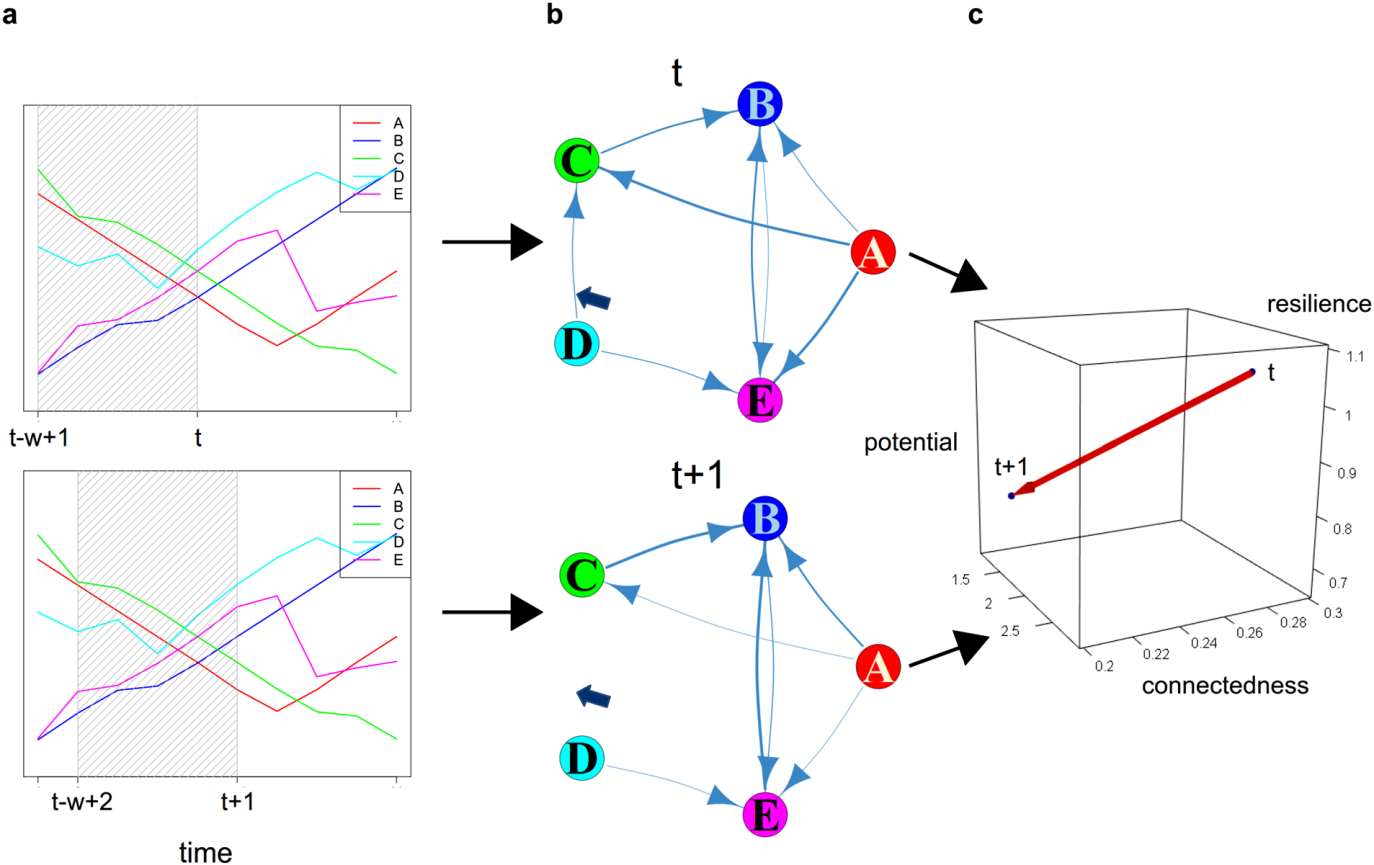
Schematic representation of our quantification method. In the first step, time series of abundance data (**a**) are transferred into networks of information transfer (**b**). In the second step, the three systemic variables (**c**) are computed on basis of the networks. The figure depicts the window shifting method.

We created the R package QtAC in order to enable a straightforward application of our method^27^. The package comprises all functions required to compute a system’s course through the adaptive cycle and to visualize the results.

Figure 2 illustrates the key idea of our approach. Figure 2a shows randomly generated abundances of five components (A,B,C,D,E). To estimate the position of this small system within the adaptive cycle at time *t* and $t+1$, we estimate transfer entropy for all pairs of components based on the samples within the window $(t-w+1, \dots , t)$ and $(t-w+2, \dots , t+1)$, respectively, and test for significance. This results in two inferred interaction networks shown in Fig. 2b. Using these networks, we can compute potential, connectedness and resilience at these two points in time. Figure 2c depicts the shift the system has made in the coordinate system spanned by the three characteristic variables.

The decrease in resilience from *t* to $t+1$ mainly follows from loosing the edge $D\rightarrow C$ at $t+1$. With component *D* being connected with the rest of the system by one edge, only, the system becomes more vulnerable, since perturbation of the edge $D\rightarrow E$ would fully decouple *D* from the rest of the system. Similarly, the loss of this edge also leads to a decrease of potential. Heuristically, the more edges a system has, the more potential there is to change from one state to another. Note that the even distribution of weights also added to the system’s potential, as for example edges $A\rightarrow E$ and $A\rightarrow C$ both loose weight. The moderate decline of connectedness follows from the loss of the edge $D\rightarrow C$ as well as from the smaller capacity of the edges $A\rightarrow C$ and $A\rightarrow E$, decreasing the overall total edge weight.

## Results

In the following, we present three different case studies. First, we provide an example based on a simulation of the Tangled Nature Model (TNM)^10^. The TNM models a system of interacting (abstract) ‘genotypes’ and has been shown to generate emergent patterns of stability followed by periods of change. This example serves as a validation of our choice of definitions. Therefore, we do not make use of the interaction structure underlying the model. As an outcome of the model we solely use the abundances of the genotypes. As a second example, we consider three European national economies^28^, demonstrating the power of the approach in explicitly comparing complex systems. In order to demonstrate the ability of the method in identifying drivers of system development, we provide a third example of a plant ecosystem being managed under a defined intervention scheme^29^.

### Capturing interaction patterns

The Tangled Nature Model (TNM)^10^ is an abstract model of co-evolutionary interactions. It simulates the development of a community of genotypes, being represented by binary vectors of a fixed length. Each of the genotypes provides an opportunity for individuals of this type to be born, mutate, reproduce, or die. The probability for these events is based on an underlying interaction pattern among the genotypes which is randomly generated at the beginning of the simulation. The model yields time series of abundances of representatives of genotypes, each of which will be considered as an agent of a system. Since there is no metabolism being modelled, it is the interactions only, which drive the dynamics of the simulation. Therefore, potential does not differ from connectedness in this system. Hence, the TNM provides an ideal testing scenario to validate our concept of connectedness and resilience since the model has been shown to capture alternating phases of stability and change^10^.

We chose a genome space of size 32 and simulated an evolution of 5000 cycles. The data is provided in the supplementary material of this paper. For the simulation, we used the code provided by Jensen and Palmieri^10^. See Suppl. Table 3 for a list of the parameters used. During the whole simulation, 24 different genotypes occurred.

**Figure 3:**
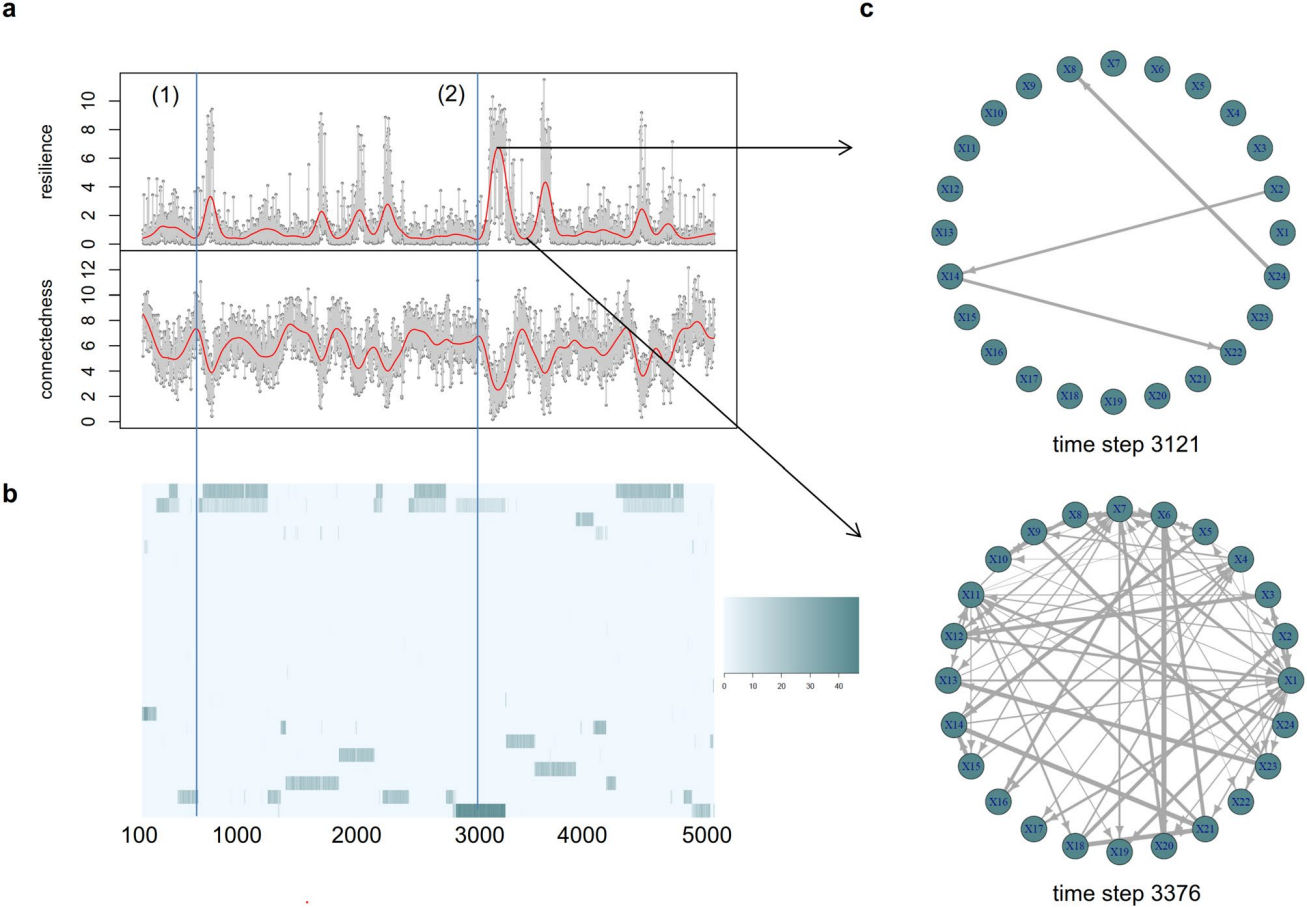
Information theoretical analysis of a Tangled Nature Model system. (**a**) Resilience and connectedness of the system (genome size of 32) over 5000 time steps. (**b**) Occupation of genome space. The blue lines mark two different kinds of breakdowns. After (1), the composition of the genome space changes, breakdown (2) happens in the middle of a stable phase. (**c**) Networks of information transfer of the simulated system at time steps 3121 and 3376.

Considering the development of connectedness and resilience of the simulated system, one can immediately observe an antagonistic cyclic behavior (Fig. 3a) with connectedness peaking while resilience drops and vice versa. This behavior perfectly matches with the variables’ anticipated development. According to the adaptive cycle metaphor, high connectedness indicates a phase of conservation which thus implies high rigidity of the existing structure of interactions. Being in such a phase, a system typically lacks flexibility exhibiting low robustness to unpredicted perturbations. Hence, our measures of resilience and connectedness capture the intrinsic dynamics described in the metaphor.

Taking a closer look at breakdowns, two different kinds of breakdown can be identified (see Fig. 3b). One type of structural change occurs right before the composition of the genome space changes (1), while the other arises in the middle of a compositionally stable phase (2). The first case literally follows the adaptive cycle metaphor: release and a subsequent phase of reorganization are characterised by structural changes of the system. The second type seems to be non-intuitive at first glance. However, an $\Omega$-phase does not need to result in compositional changes. A structural breakdown of organisation can also manifest itself in internal reorganization. The external structure of the system thereby appears to remain unaffected. Examples of such an internal crisis are abundant in both ecology and socio-economical systems.

A clear pattern in the development of the information networks reflects the different phases of the cycle. As exemplarily shown by the network at time step 3121 (see Fig. 3c), the network of information transfer does only have a few edges in times of higher resilience. This is typically the case during phases of release and reorganization. From step to step, these edges quickly change and do not exhibit any pattern of continuity. In contrast, in times of lower resilience, hence during phases of exploitation and conservation, the network reveals many edges (see, e.g., the network at time step 3376 in Fig. 3c). Here, the edges range from very low-weighted edges, representing weak transfers of information, to heavy-weighted edges, reflecting strong transfers and thereby more closely connected components. This confirms the description of the four phases by Gunderson and Holling^1^. Recall that the transition from *K*- to $\Omega$-phase is characterized by connections being broken, low connectivity among variables, and unexpected associations and recombinations appearing (see Ref.^1^, p. 45pp).

### Comparing systems

One major advantage of using transfer entropy rather than physical flows for capturing interaction lies in the potential to compare systems independently of their physical instantiation. To demonstrate this feature, we analysed economic data from three different European countries.

Recall that in the second decade of the 21st century, Europe has been shaken by a financial crisis. Considering each country as an economic system, classical economic variables such as export, import and gross national product can be considered as agents. We complemented this set of monetary variables with logistic variables such as the amount of goods transported by road or by railway to incorporate logistic capacity. Variables such as final consumption expenditure of households, motorisation rate, or people at risk of poverty or social exclusion reflect the prosperity of a country. Additionally, environmental indicators such as greenhouse gas emissions are considered. Our data set comprises yearly quantities of the economic variables from 2004 to 2015, being obtained from the Eurostats database^28^. The data is provided in the supplementary material. See Suppl. Table 1 for a complete list of the variables used.

In the aftermath of the crisis, European countries have been split into two major classes, i.e., the Northern countries representing creditor states, and Southern/peripheral countries, representing the debtor states. These two groups drifted into the crisis under different prerequisites and consequently played unequal roles. As a representative of the former group, we chose Germany. For the debtor group, we chose Greece and Italy. While Greece is a special case since restructuring of dept became necessary to overcome crisis^30^, Italy represents a Southern state without international control during the study period.

**Figure 4:**
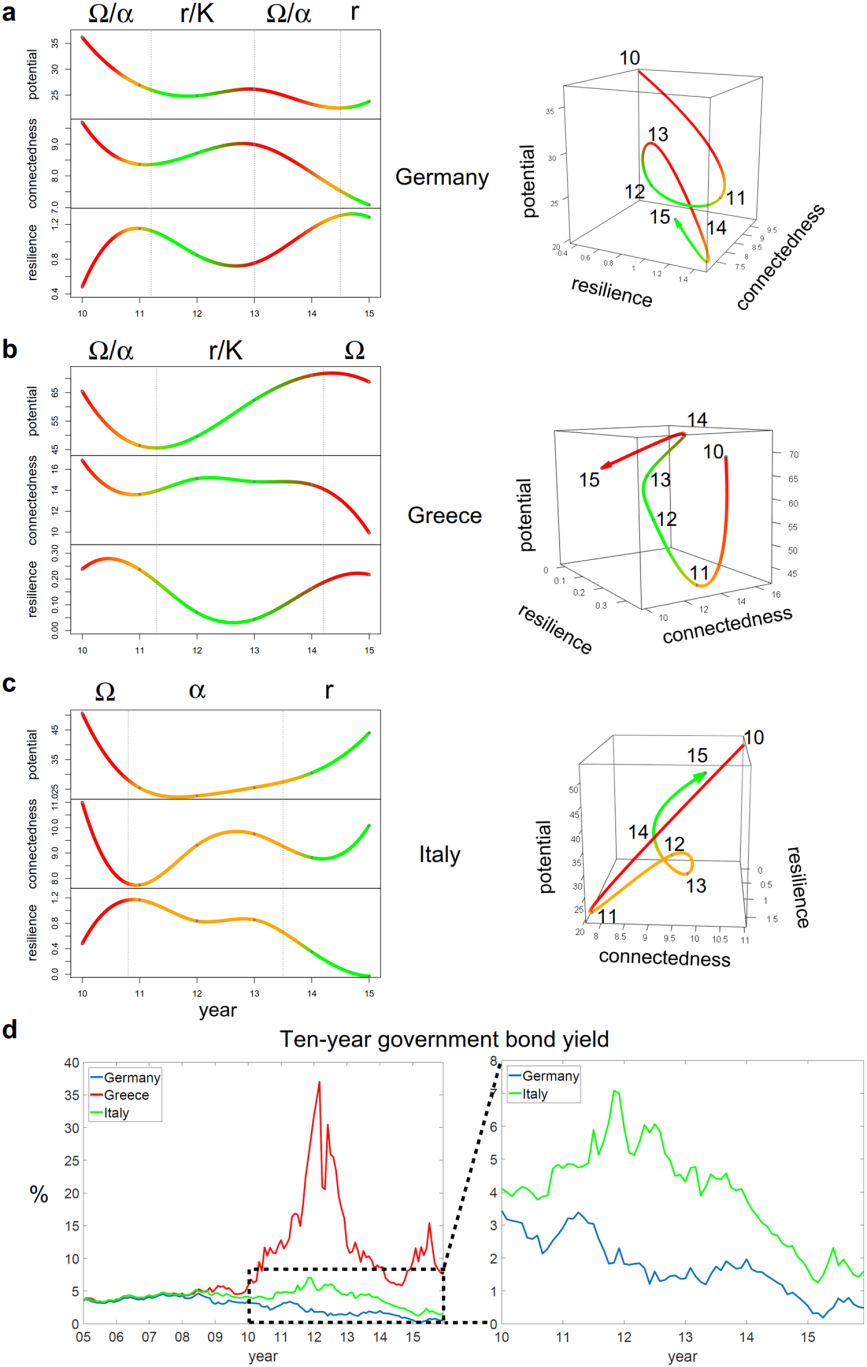
Information theoretical analysis of European countries during the Euro crisis. (**a**–**c**) Development of potential, connectedness, and resilience of Germany, Greece and Italy considered as economic systems. (**d**) Ten-year government bond yield of Germany, Greece and Italy^36^. The labels mark the month January of the respective year.

Figure 4a shows a classical $\Omega$-phase during 2010–2011 for Germany. A breakdown in potential and connectedness is accompanied by an increase in resilience. This phase of economic decline can also be see at the financial market. Note that an increase in returns from ten-year government bonds (see Fig. 4d) reflects decreasing trust in the governments capacity to act at the onset of the crisis^31^.

From 2011 to 2013, potential and connectedness recover, indicating a clear *r*/*K*-phase. This increase goes along with a loss of resilience, leading to a second breakdown in 2013. These phases can also be followed in the development of government bond yields, decreasing from 2011 to 2013 followed by another increase until the first months of 2014. From 2014 to 2015, the yields decreased again with the system starting into another $\alpha$-phase. Overall, Germany runs through a complete adaptive cycle during these years. This well matches the fact that Germany has not been hit by the Euro crisis so hardly. Like most of the Northern countries, Germany had a finance surplus when the global financial crisis started, which allowed the country to take-in the role of a creditor^30^.

From a system point of view, the development of the Greek economy shows a similar behaviour (Fig. 4b). After a breakdown in 2010–11, an *r*/*K*-phase can be identified in 2011–2013 with the year 2014 indicating another change to come. Considering yields of ten-year government bonds (Fig. 4d), the conclusion would be partially opposite. The beginning of the *r*/*K*-phase from 2011 to 2012 is coming along with a steep increase in yields, showing the fact that the markets lost their trust in the Greek economy. Recall that at the end of 2009, Greece’s credit ratings have been downgraded after the Greek government had disclosed an extraordinary high budget deficit^32^. In the following, financial assistance programs have been approved (e.g. “Six-Pack of reforms” in December 2011^32^) aiming at the stabilization of the economy. From our system perspective, these external actions seem to have had a stabilizing and growth encouraging effect from 2011 onwards. Thus, through the intervention of the European community, actual market development as being captured by economic performance indicators and thereby by our three systemic variables has been decoupled from the financial assessment of the country’s economy, as being reflected in governmental bonds.

After a second financial assistance package in March 2012^32^, potential and connectedness of the country continue to increase. While this rise lasts until 2014 for potential, connectedness already declines from 2012 on. This ’atypical’ behaviour might indicate the fact that the exploitation phase has been triggered externally. Due to the formation of a new government, European payments were suspended from August 2014 to July 2015^33^. With internal control taking over again in that period, a second breakdown can be seen, which now is indeed being reflected in a second peak in governmental bond yields.

Italy exemplifies a debtor state without international intervention. From 2010 to 2012, potential and connectedness overall decrease along with an incline in resilience (Fig. 4c). At the same time, yields of ten-year government bonds increased (Fig. 4d). During the following three years, yields decreased again. A particularly strong decline can be observed between 2014 and 2015, leading to a local low at the beginning of 2015. Indeed, 2014 is the year where both potential and connectedness start increasing strongly while resilience decreases. Here, we identify a classical *r*/*K*-phase. In summary, the systemic analysis well matches the economic situation of the country. In contrast to Greece, Italy could avoid a bailout. Nevertheless, structural reforms and austerity measures progressed slowly^34^. The country faced massive increases in unemployment and poverty, and populist and anti-establishment parties were strengthened in the sequel^30^.

### Identifying drivers of system change

With our third case study, we return to ecosystem development. The example of the development of a plant community in the prairie-forest ecotone of Eastern Kansas under intervention demonstrates the use of the entropy approach to identify drivers of change. The data underlying this case study was collected in the course of a succession experiment in an experimental plot in Kansas every June from 2002 to 2015. We excluded those species which appeared at most once during the observation period, covering less or equal ten percent of the experimental plot. For details on the experiment and data collection, see Ref.^29^, Exper. 1, Unit 13. For a complete list of the plant species and their features used in the ecological interpretation see Suppl. Table 2.

Our study period ranges from 2007 to 2015. The system comprises 39 species being present in the study plot, including grasses, forbs, shrubs, trees, and vines. Their relative abundance has been measured every year in June. In 2008, 2011 and 2014, intentional spring burns have been executed shortly before data collection.

**Figure 5:**
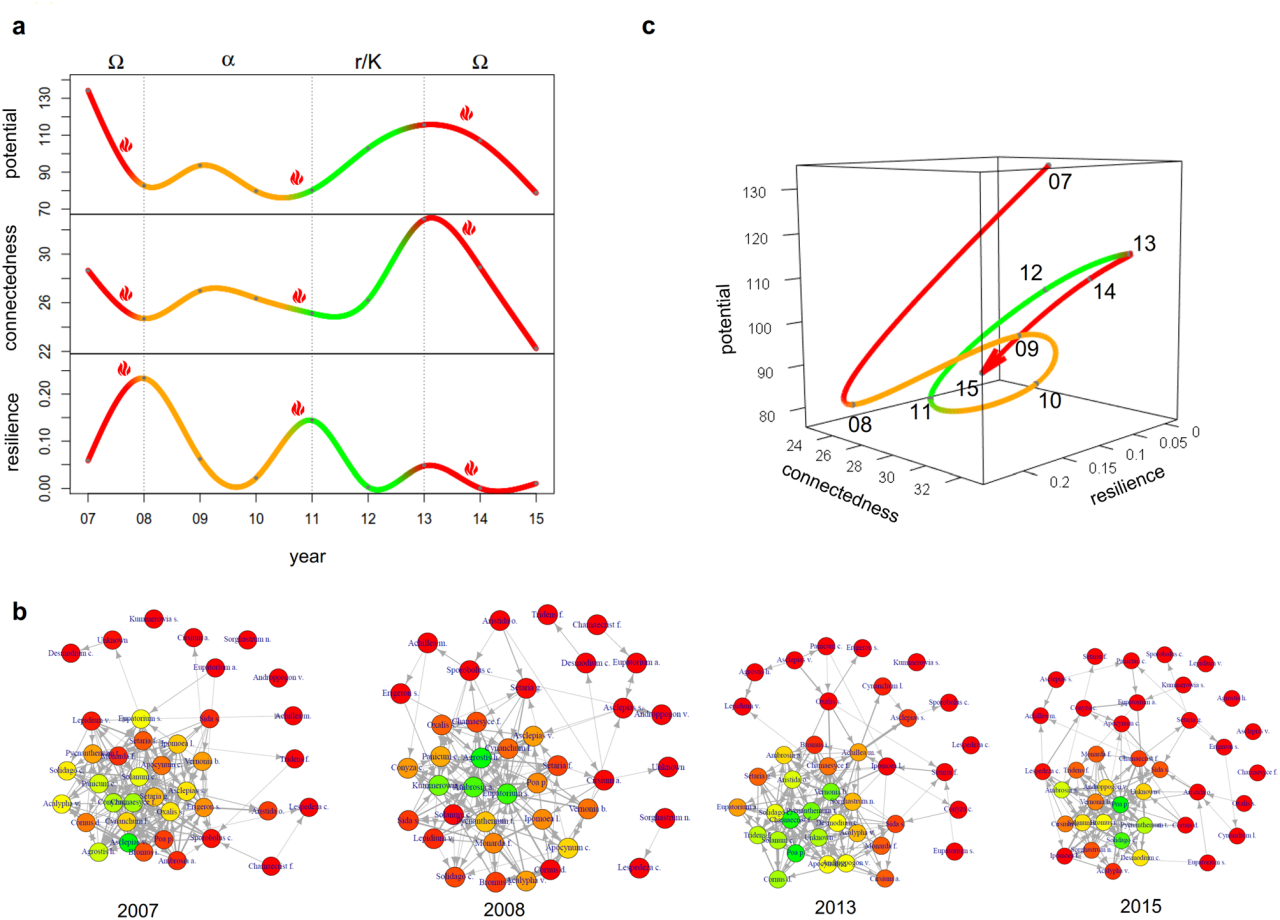
Information theoretical analysis of a plant community being exposed to human intervention in Kansas. (**a**) Potential, connectedness and resilience of the community of plants in the prairie-forest ecotone of Kansas. The labels mark the month June of the respective year. The flames indicate the times of the spring burns. (**b**) Networks of information transfer of the plants’ community in 2007, 2008, 2013, and 2015. The nodes are coloured according to their eigencentrality. Red marks low values of eigencentrality, orange eigencentrality of medium height, and green high values of eigencentrality. (**c**) Three-dimensional plot of potential, connectedness, and resilience of the plant community.

The three characteristic variables indicate a clear division into four phases (Fig. 5a,c). At the beginning, we can identify a classical $\Omega$-phase 2007–08. Supposedly, this breakdown results from the preceding spring burn. Within the next two years, the system is not able to considerably regain potential or connectedness. At the same time, resilience monotonously decreases. This changes abruptly with the spring burn in 2011, which naturally leads to a strong increase in resilience. Now the three variables are in a classical initial situation for another *r*/*K*-phase. The following climax phase leads to a peak in potential and connectedness in 2013. A subsequent breakdown interestingly occurs a year before the third intervention in 2014. Furthermore, resilience does not recover during this period, as would have been expected from the adaptive cycle metaphor. We will come back to this point later.

**Figure 6:**
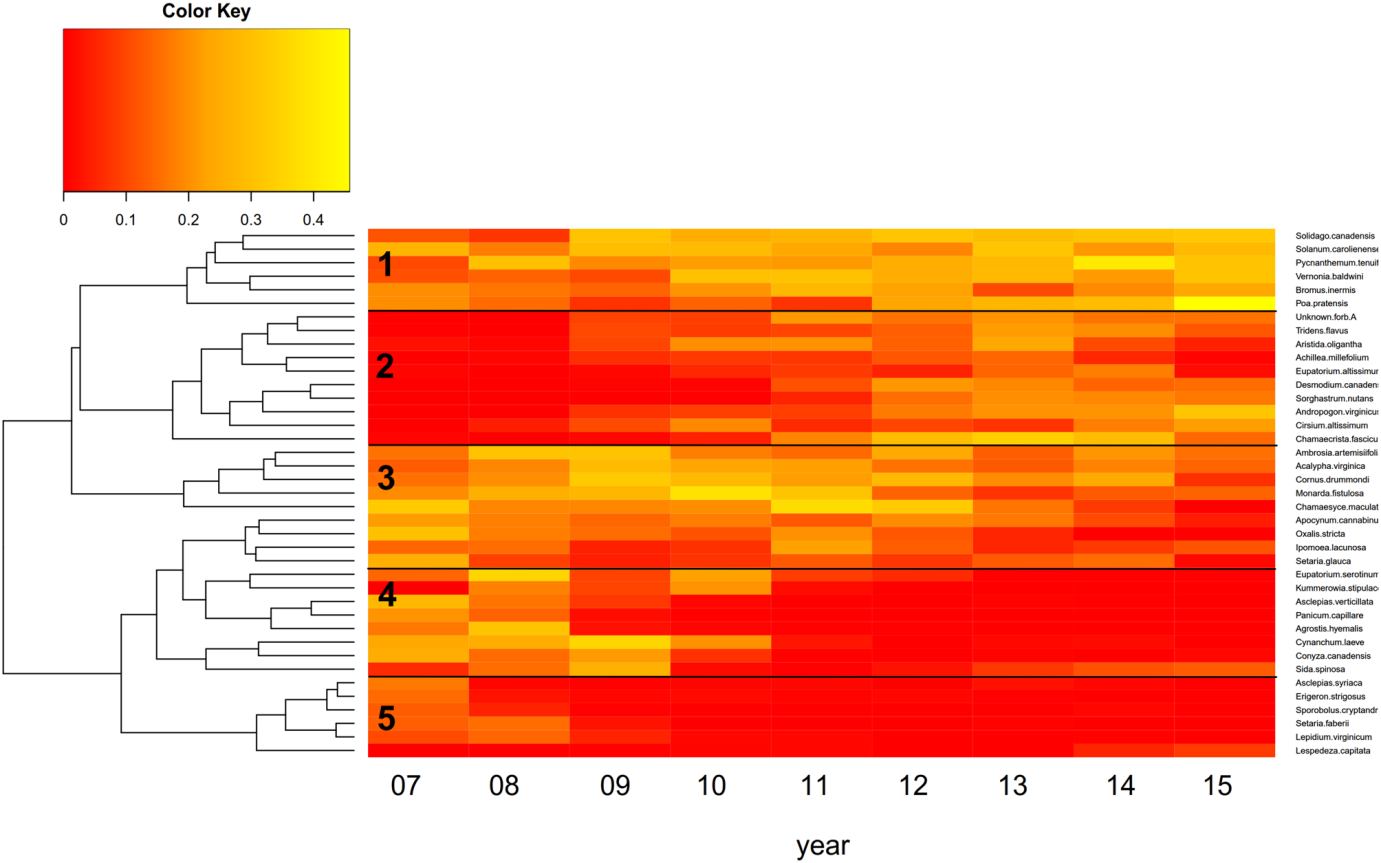
Heatmap showing the eigencentrality of the species in the networks of information transfer from 2007 to 2015. The species are divided into five groups according to the pattern of their eigencentrality development.

In order to identify drivers of change, let us consider the networks of information transfer more closely (see Fig. 5b). At times of high connectedness (i.e. in 2007 and 2013), the network is characterised by a large number of nodes showing high eigencentrality. In contrast, there are fewer nodes of high centrality in networks showing lower connectedness (i.e. in 2008 and 2015). At the same time the number of loosely connected nodes increases. Thus, the large strongly connected core in 2007 and 2013 partly dissolves with the system collapsing. The structure of the core appears to be closely related to the dynamics of the system. Partitioning the set of nodes according to their centrality over time (see Fig. 6), five groups of plants can be identified. Group 2 is characterized by high centrality values at the end of the study period, only, whereas Group 4 shows the opposite behaviour. Group 1 and Group 3 exhibit higher centrality all over the study period, with the former being lower at the beginning and the latter towards the end. Finally, Group 5 comprises species which are of small centrality almost all over.

Taking a closer look at the ecological features of the plants in either group (see Suppl. Table 2), this pattern can be explained. The first two groups contain considerably more perennial and fire tolerant species compared with the other groups. Hence, these species should be expected to gain importance throughout the sequence of fire interventions. Furthermore, the species in the first and third group tend to have higher growth rate and higher vegetative spread rate. Therefore, these features should favour a long-term central role under repeated interventions. Comparing both properties, being perennial and fire tolerant (Group 2) seems to be beneficial over time, while higher reproduction capabilities (Group 3) cannot cope with repeated fire intervention on a longer term. Note that it is much harder to infer such findings from species abundance counts, only.

**Figure 7:**
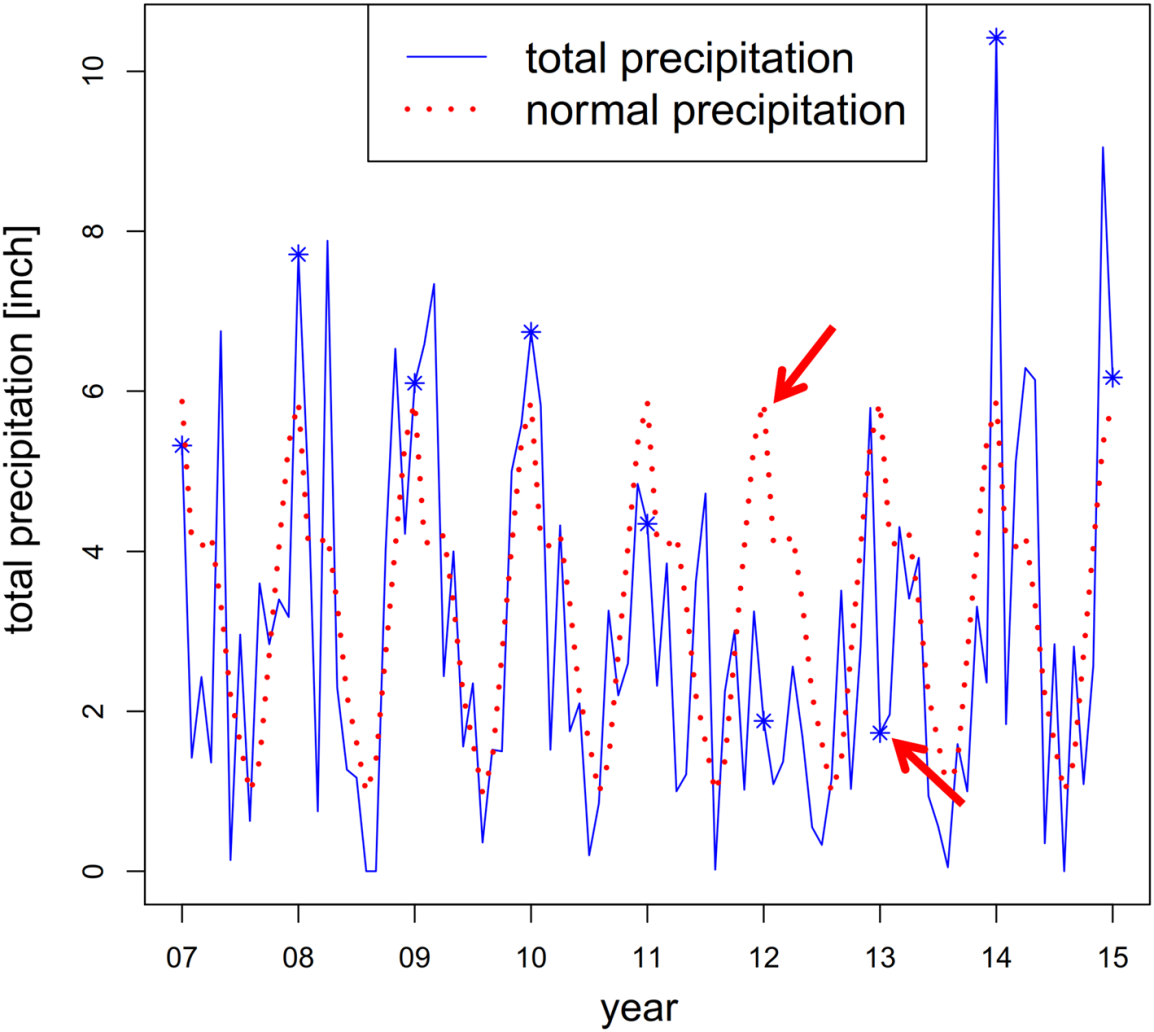
Precipitation in Lawrence. The plot shows the total precipitation in Lawrence, Kansas, where the experimental plot is located. In comparison, the normal precipitation for this area is shown^38^. The stars label the month June of the respective year.

Finally, let us return to the atypical breakdown in 2013. Regional climate data shows that total precipitation has been extraordinary low in 2012. In the first half of 2013, the situation seems to relax slightly. But in June, at the time data was collected, total precipitation again dropped significantly below normal (Fig. 7). Thus, the plants have been exposed to an unusual high drought stress during the *r*/*K*-phase. In addition, the central core during this phase mainly consists of species being less drought tolerant. Hence, the plant system has not been well adapted to the particular environmental conditions during these years. The breakdown starting in 2013 is thus likely caused by increased vulnerability of the plants. This last observation nicely shows the difficulty in analysing ecosystems under experimental intervention scenarios. Effects due to the planned disturbance always also interact with systemic effects under natural conditions. Nevertheless, our approach allows to dissect the two types of perturbation.

## Discussion

We presented a method to quantify maturation of general complex systems, independently of their concrete realization. Our method allows to include measurements of different units and proportions into the analysis of one or several systems. As a basis it only requires the most basic information about an underlying system being available, namely time series of its components’ states. This constitutes a significant progress since comparable analyses so far have always relied on specific knowledge about the system to be available. More general approaches require to capture physical flows in order to apply information theoretic measures^11,35^, which is experimentally demanding. Our method overcomes these restrictions by the twofold application of information theory. We utilize its generic nature to quantify the three systemic variables. The generality of our approach results from targeting information transfer instead of physical flows.

The estimation of transfer entropy is a non-trivial task and requires appropriate statistical estimators. In order to obtain statistically reliable results, a certain length of time series is needed. The KSG estimator that we use is optimized for short time series^21^. Nevertheless, we recommend not to use series shorter than five time points for the estimation of one network. Hence, in order to analyse a system at a time point, one needs abundance data of at least four preceding time points. Note that there is a trade-off in choosing the window size for estimating transfer entropy. While larger windows may provide higher statistical reliability, they also increase the impact of past events onto the estimation. A natural choice can be made by taking into account the estimated length of the memory of the Markov process.

There are no general limitations concerning the number of components of the system. Obviously, there is not much sense in analysing systems of less than three components. For larger systems, summary statistics of large networks might be used for interpretation. In general, the choice of the proper scale, i.e. the number of relevant components, is an important aspect in analysing complex systems which should be chosen with care.

Our case studies accentuate our choice of measures, revealing the expected interplay between potential, connectedness and resilience. Furthermore, we demonstrated that these measures are reasonable as being confirmed by consulting background knowledge about the systems being studied. Overall, our analyses support the concept of the adaptive cycle. We were able to detect the predicted cyclic behavior of potential, connectedness and resilience in all systems being considered.

At the same time, the case studies also confirm the conceptual character of the adaptive cycle. As being expected, the four phases do not always occur in the idealistic manner as being described in the metaphor. Some of the details that Gunderson and Holling draw on cannot be supported by our study. For example, we cannot confirm a generally short duration of the $\Omega$- and $\alpha$-phase compared to the r- and K-phase. The duration of the phases rather results from the interplay of environmental dynamics with the internal evolution of the system. Such an interplay cannot be expected to always result in fixed patterns of temporal dynamics.

Our method can not be used in a black box fashion. It clearly depends on a careful choice of parameters to be made by the user. In particular, less educated choices might crucially influence estimation of transfer entropy. Ideally, these choices are made with some a priori understanding of the timescale being relevant for the development of the system. In case there is no such knowledge available, refined estimation techniques can be applied (see^24^ for details).

In conclusion, our approach is indeed capable of analysing systems independently of their specific realization. The approach is widely applicable bearing no inherent interpretation such as ecosystem or economic theory. The approach does leverage the metaphor of the adaptive cycle to an operational level. Future work lies in improving scalability of the approach. Furthermore, managing complex systems relies on experience and familiarity with the system’s dynamics. Thus, combining our approach of analysis with proper tools for simulation can provide a powerful tool for understanding sustainability and guiding system management.

## Supplementary information

Supplementary Information 1.

Supplementary Information 2.

Supplementary Information 3.

## Data Availability

The dataset generated during the analysis of the Tangled Nature Model and the countries’ economic data analysed in the Euro crisis case study are provided in the supplementary material of this paper. Metadata of this case study was collected at^36^. The main dataset used in the Kansas case study is available in the EDI repository^37^. Metadata on plants’ features is listed in Suppl. Table 2. The precipitation data can be found at^38^.
