# Supplementary material for: Computing the adaptive cycle
Source: Sci Rep. 2020 Oct 23;10:18175. doi: 10.1038/s41598-020-74888-y (PMC7584630; doi:10.1038/s41598-020-74888-y)
Supplement: Supplementary file 2 — Supplementary Information 2. [file 41598_2020_74888_MOESM2_ESM.pdf]

Computing the Adaptive Cycle

Supplementary

Wolfgang zu Castell[1,2] **and Hannah Schrenk**[1,*]

[1]Helmholtz Zentrum München, Ingolstädter Landstraße 1, D-85764 Neuherberg
[2]TUM München, Boltzmannstraße 3, D-85748 Garching bei München
[*]hannah.schrenk@helmholtz-muenchen.de

ABSTRACT

Gunderson's and Holling's adaptive cycle metaphor provides a qualitative description of the development of a dynamically evolving complex system. According to the metaphor, a complex system alternately passes through phases of stability and predictability and phases of reorganization and stochasticity. So far, there have been no attempts to quantify the underlying notions in a way which is independent of the concrete realization of the system. We propose a method which can be applied in a generic way to estimate a system's position within the adaptive cycle as well as to identify drivers of change. We demonstrate applicability and flexibility of our method by three different case studies: Analyzing data obtained from a simulation of a model of interaction of abstract genotypes, we show that our approach is able to capture the nature of these interactions. We then study European economies as systems of economic state variables to illustrate the ability of system comparison. Finally, we identify drivers of change in a plant ecosystem in the prairie-forest. We hereby confirm the conceptual dynamics of the adaptive cycle and thus underline its usability in understanding system dynamics.

A. Review of the adaptive cycle

In their adaptive cycle metaphor[1], Gunderson and Holling describe the evolution of a complex system using three comprehensive system properties: potential, connectedness and resilience[1]. Each of them represents a fundamental aspect of system adaptation. The *potential* of a system encompasses the capacity a system has at its disposal to react to future changes. As a consequence of adaptation to environmental conditions and exploitation of resources, a system tends to increase its internal level of organization, often accompanied by an increase in rigidity and a decrease in flexibility. This is what is described by *connectedness*. It is an overall quantity of flexibility or rigidity. The third variable captures a momentum of change. Gunderson & Holling define resilience as "*the magnitude of disturbance that can be absorbed before the system changes the variables and processes that control behavior*" [1, p.28].

Envisioning an environment of largely unexplored resources, a system will first tend to make use of these resources without any need of higher efficiency. Such environmental conditions are typically encountered together with less stability, since the environment has not yet been shaped. In ecology, this is the opportunity for r-strategists[2]. Following this terminology, the exploitation phase is also called the *r-phase* of system development. Connectedness is still low, potential first needs to be build-up, while resilience is typically high.

Starting to make use of the resources, the system will increase its inner level of organization. Expertise is generated and through the action of the system new opportunities (i.e. *niches*[3]) are generated, contributing to an increase in optimality with respect to resource utilization. The conservation phase is termed *K-phase*. Indeed, in ecological terms, K-strategists characterize the system within this period.

However, while increasing levels of optimization make better use of resources, the increased level of internal organization and specialization has to be sustained. Organization is not free of cost and therefore requires a share of the resources being captured. This typically leads to a decrease in resilience. Eventually, the probability of encountering an external event the system can not immediately cope with increases. "*In the cases of extreme and growing rigidity, all systems become accidents waiting to happen*" [1, p.45]. Sooner or later, a presumably small trigger then leads to a breakdown of the current organization in the following Ω-*phase*. Resources being captured by the system will be released and a phase of reorientation is initiated.

The reorganization phase is also named α-*phase*, standing at the beginning of a new cycle. Various opportunities will be exploited, some of them leading to new developments, while others quickly disappear. A system needs to cope with a high level of stochasticity. During this period, the system is being driven by changing environmental conditions, rather than being capable of shaping the environment towards its own favor. New components might enter the system, while others get lost. During breakdown (Ω-phase), resilience increases while potential gets lost.

The phases of exploitation and conservation have long been established in the theory of ecological succession. As an outcome of an interdisciplinary discussion on general properties of evolving systems, Gunderson & Holling added the more stochastic period of the cycle being represented by release and reorganization. Representing phases of change, these stages are harder to be observed. Whatever makes us as an observer be interested in perceiving a group of interacting agents as a system, will typically be less explicit during such times of trial and error[4].

B. Data of the Eurostat case study

Quantity	Unit
gross domestic product	mio €
exports of goods and services	mio €
imports of goods and services	mio €
volume of freight transport	index (2010 = 100)
volume of passenger transport	index (2010 = 100)
greenhouse gas emissions	index (1990 = 100)
pollutant emissions from transport	nitrogen oxides
greenhouse gas emissions intensity of energy consumption	index (2000 = 100)
energy consumption of transport	index (2000 = 100)
gross value added of the agricultural industry	mio €
railway passenger volume	mio
goods transported by railway	thousands of tonnes
motorisation rate	cars per 1000 inhabitants
goods transported by road	thousands of tonnes
air transport of passengers	passengers
air transport of goods	tonnes
final consumption expenditure of households	mio €
people at risk of social poverty or exclusion	thousands of people
current account transactions - balance	mio €

Supplementary Table 1. Quantities and the corresponding unities used in the Eurostat case study.

C. Species and metadata of the Kansas case study

Species	Growth Habit	Duration	Fire Tolerance	Growth Rate	Drought Tolerance	After Harvest Regrowth Rate	Vegetative Spread Rate
Acalypha,virginica	f	a	none	rapid	medium	slow	none
Achillea,millefolium	f	p	high	moderate	medium	moderate	slow
Agrostis,hyemalis	g	p	high	moderate	low	moderate	moderate
Ambrosia,artemisiifolia	f	a					
Andropogon,virginicus	g	p	high	slow	high	slow	none
Apocynum,cannabinum	f	p	none	moderate	medium	slow	none
Aristida,oligantha	g	a					
Asclepias,syriaca	f	p	burning stimulates				
Asclepias,verticillata	f	p					
Bromus,inermis	g	p	high	moderate	medium	slow	rapid
Chamaecrista,fasciculata	f	a	none	rapid	medium		none
Chamaesyce,maculata	f	a					
Cirsium,altissimum	f	b					
Conyza,canadensis	f	a,b	low	rapid	low	slow	none
Cornus,drummondi	t,s	p	low	rapid	low		rapid
Cynanchum,laeve	f,v	p					
Desmodium,canadense	f	p	burning stimulates		low		
Erigeron,strigosus	f	a,b,p	low	moderate	medium	slow	none
Eupatorium,altissimum	f	p					
Eupatorium,serotinum	f	p					
Ipomoea,lacunosa	f,v	a					
Lepidium,virginicum	f	a,b,p					
Kummerowia,stipulacea	f	a	medium	rapid	medium	slow	none
Lespedeza,capitata	f	p	high	slow	high	slow	none
Monarda,fistulosa	f,ss	p	none	moderate	no	slow	slow
Oxalis,stricta	f	p			high		
Panicum,capillare	g	a			high		
Poa,pratensis	g	p	high	moderate	low	moderate	rapid
Pycnanthemum,tenuifolium	f	p		high	medium		rapid
Setaria,faberii	g	a					
Setaria,glauca	g	a			medium	moderate	rapid
Solidago,canadensis	f	p	high	rapid	medium	moderate	
Sida,spinosa	f,ss	a,p					
Solanum,carolienense	f,ss	p					
Sorghastrum,nutans	g	p	high	moderate	medium	moderate	moderate
Sporobolus,cryptandrus	g	p	medium	moderate	high	slow	none
Tridens,flavus	g	p	high	moderate	high	rapid	none
Unknown,forb,A							
Vernonia,baldwini	f	p					

Supplementary Table 2. Features of the species occurring in unit 13 of the succession experiment in Kansas[5]. The following abbreviations are used: f - forb/herb, g - graminoid, t - tree, s - shrub, ss - subshrub, v - vine, a - annual, p - perennial, b - biennial.

D. Parameters of the Tangled Nature Model simulation

initial population size	$N(0) = 2$
coupling parameters	$\Theta = 0.3$ and $c = 14.3$
reproduction parameter	$\mu = 0.2$
mutation probability	$p_{mut} = 0.001$
annihilation probability	$p_{kill} = 0.02$

Supplementary Table 3. Parameters used in the Tangled Nature Model simulation.

E. Resilience in the language of spectral graph theory

Spectral graph theory enables us to study graphs using algebraic tools by considering graph-related matrices and their eigenvalues. Speaking about undirected graphs, there is great interest in the Laplacian matrix and its eigenvalues[6,7]. Our definition of resilience is based on an analogue of the Laplacian for directed graphs.
In the following, we will introduce this matrix and some of the properties of its eigenvalues in the classical, undirected case to make clear why it inspired us to our definition. Eventually, we will present our analogues of the Laplacian matrix in the directed case and define resilience.

Let $G=[V,E]$ be a weighted, undirected graphs with $N \geq 1$ nodes and weight function $w\colon E \to \mathbb{R}_+$. We call

$$d_u = \sum_{v\in V} w(u,v)$$

the *degree* of a node u. The graph's *adjacency matrix* is defined as

$$A(u,v) = w(u,v)\ \forall u,v \in V,$$

its *degree matrix* as

$$D(u,v) = \begin{cases} d_u, & \text{if } u=v \\ 0, & \text{otherwise.} \end{cases}$$

From now on, we will follow the convention $D^{-1}(u,u)=0$ if $D(u,u)=0$. Multiplying $(D-A)$ with $D^{-\frac{1}{2}}$ from both sides, yields the *Laplacian matrix* of G:

$$\begin{aligned} L &= D^{-\frac{1}{2}} \cdot (D-A) \cdot D^{-\frac{1}{2}} \\ &= I - D^{-\frac{1}{2}} \cdot A \cdot D^{-\frac{1}{2}} \end{aligned}$$

Therefore, we have

$$L(u,v) = \begin{cases} 1-\frac{w(u,u)}{d_u}, & \text{if } u=v \text{ and } d_u \neq 0 \\ \frac{-w(u,v)}{\sqrt{d_u \cdot d_v}}, & \text{if } u \neq v \text{ and } d_u \neq 0,\, d_v \neq 0 \\ 0, & \text{otherwise.} \end{cases}$$

As the Laplacian matrix is symmetric, all of its eigenvalues are real and non-negative. A short computation shows that 0 is always an eigenvalue of L. Let $\sigma_0 = 0 \leq \sigma_1 \leq \cdots \leq \sigma_{n-1}$ be the spectrum of L. The multiplicity of the eigenvalue 0 is strongly connected with the topology of the corresponding graph G: It is equal to the number of unconnected components of G[6].

Our interest lies on the smallest non-trivial eigenvalue σ_G of the Laplacian. It can be understood as a measure of "how close" G is to breaking into another unconnected component. If σ_G is close to 0, a slight change of the Laplacian can shift it to 0. Hence, we know that a little disturbance of the graph, like the deletion of an edge with small weight, can generate a new unconnected component. Whereas, if σ_G is high-valued, a great disturbance, like the deletion of many or heavy-weighted edges might be needed to create a new component.
Let us recall Holling's definition of resilience: "*the magnitude of disturbance that can be absorbed before the system changes the variables and processes that control behavior*" [1, p.28]. This change is a consequence of the system's impossibility to get back to its former state. Thinking of the system as a network of transfers, the split up of a new component inhibits any flow between the new component and the rest of the graph. There are no alternative pathways to compensate the lost edges and thereby restore these transfers. The system has moved to a new state. These thoughts inspired us to choose σ_G as mathematical definition of Holling's resilience. But the classical form of σ_G is insufficient for us as it is only defined for undirected graphs. We are interested in directed graphs, considering the direction of transfers/flows within a system as crucial for its identity. Therefore, we need to transfer these notions to the case of directed graphs. Several versions of the Laplacian matrix of a directed graph exist in literature[8],[9]. Inspired by those, we define two Laplacian matrices associated to a directed graph, L_{in} and L_{out}.

Let $G=[V,E]$ be a weighted, directed graph with $N \geq 1$ nodes and weight function $w\colon E \to \mathbb{R}_+$. We call

$$d_{u,out} = \sum_{u\to v} w(u,v)$$

respectively

$$d_{u,in} = \sum_{v \to u} w(v,u)$$

the *out*- respectively *in-degree* of a node u. Let $c > 0$ be a constant. We define the *directed Laplacian matrices* of G by

$$L_{out}(u,v) = c \cdot \begin{cases} 1 - \frac{w(u,u)}{d_{u,out}}, & \text{if } u = v \text{ and } d_{u,out} \neq 0 \\ \frac{-w(u,v)}{\sqrt{d_{u,out} \cdot d_{v,out}}}, & \text{if } u \neq v \text{ and } d_{u,out} \neq 0,\, d_{v,out} \neq 0 \\ 0, & \text{otherwise} \end{cases}$$

and

$$L_{in}(u,v) = c \cdot \begin{cases} 1 - \frac{w(u,u)}{d_{u,in}}, & \text{if } u = v \text{ and } d_{u,in} \neq 0 \\ \frac{-w(u,v)}{\sqrt{d_{u,in} \cdot d_{v,in}}}, & \text{if } u \neq v \text{ and } d_{u,in} \neq 0,\, d_{v,in} \neq 0 \\ 0, & \text{otherwise.} \end{cases}$$

Analogously to the undirected case, the Laplacian matrices have a relation to the adjacency matrix A and the directed degree matrices

$$D_{out}(u,v) = \begin{cases} d_{u,out}, & \text{if } u = v \\ 0, & \text{otherwise} \end{cases}$$

and

$$D_{in}(u,v) = \begin{cases} d_{u,in}, & \text{if } u = v \\ 0, & \text{otherwise.} \end{cases}$$

A short computation shows that

$$L_{out} = c \cdot D_{out}^{-\frac{1}{2}} (D_{out} - A) D_{out}^{-\frac{1}{2}}, \quad \text{and} \quad L_{in} = c \cdot D_{in}^{-\frac{1}{2}} (D_{in} - A) D_{in}^{-\frac{1}{2}}.$$

Eventually, we can formulate our definition of resilience. To this end, we want to find an analogue of the eigenvalue λ_G defined in the previous section. But we have to be aware of the differences between the two cases. Before, we were considering symmetric matrices with real, positive eigenvalues. Now we are dealing with general matrices with complex eigenvalues. Therefore, we restrict ourselves to the absolute values of the real parts of the eigenvalues. Besides, by our definition, a directed graph has two Laplacian matrices, so our definition should respect both of them. For a system $\mathscr{V}$ considered as network with transfers $\{T_{J \to I} | (J,I) \in \mathscr{V} \times \mathscr{V}\}$, let L_{in} and L_{out} be its Laplacian matrices. We define

$$R = \min\{|\Re\sigma| : \sigma \in \mathrm{Spec}(L_{out}) \cup \mathrm{Spec}(L_{in}), \sigma \neq 0\}.$$

as the system's *resilience*.

Depending on the specific question, different choices of the standardization constant c are reasonable. A common choice is

$$c = 1.$$

However, especially when it comes to the comparison of different systems' resilience or to the development of resilience over time, standardization can be a helpful tool. Let

$$M = \max\{T^t_{J \to I} | (J,I) \in \mathscr{V} \times \mathscr{V}\}$$

be the maximal edge weight of the graph. We set

$$c = \frac{1}{M}$$

for all case studies presented in this paper. The R package QtAC offers the possibility to choose between different standardization constants.

In this paper, we use the just defined symmetrically normalized version of the Laplacian matrices. In other cases, it can be more reasonable to use

$$L_{out} = c \cdot D_{out}^{-\frac{1}{2}}(D_{out} - A), \quad \text{and} \quad L_{in} = c \cdot (D_{in} - A)D_{in}^{-\frac{1}{2}},$$

which has the advantage of a spectrum changing continuously with the edge weights. Here, common standardization choices are

$$c = \frac{1}{\sqrt{M}}$$

or

$$c = \frac{\sqrt{N-1}}{N},$$

which standardizes R with respect to the number of nodes. The R package QtAC provides both versions of the Laplacian matrices. For the case studies presented in this paper, they yield qualitatively the same results.

References

1. Gunderson, L. & Holling, C. *Panarchy: Understanding Transformations in Human and Natural Systems* (Island Press, 2002).
2. Pianka, E. On r- and k-selection. *The Am. Nat.* **104**, 592–597 (1970).
3. Odling-Smee, F., Laland, K. & Feldman, M. Niche construction. *The Am. Nat.* **147**, 641–648, DOI: 10.1086/285870 (1996).
4. Heylighen, F. Causality as distinction conservation. a theory of predictability, reversibility, and time order. *Cybern. Syst.* **20**, 361–384, DOI: 10.1080/01969728908902213 (1989).
5. Plants database of the united states department of agriculture. https://plants.sc.egov.usda.gov/java/. Accessed: 2019-04-26.
6. Chung, F. *Spectral Graph Theory* (CBMS Regional Conference Series in Mathematics Number 92, 1997).
7. Fiedler, M. Algebraic connectivity of graphs. *Czechoslov. Math. J.* **23**, 298–305 (1973).
8. Chung, F. The diameter and laplacian eigenvalues of directed graphs. *Electr. J. Comb.* **13**, DOI: 10.37236/1142 (2006).
9. Wu, C. On rayleigh–ritz ratios of a generalized laplacian matrix of directed graphs. *Linear Algebr. its Appl.* **402**, 207–227, DOI: 10.1016/j.laa.2004.12.014 (2005).
